# Primary duodenal carcinoma suspected to arise from ectopic gastric mucosa: a case report

**DOI:** 10.1186/s40792-023-01605-y

**Published:** 2023-02-13

**Authors:** Ryosuke Fukushima, Norio Kubo, Shigemasa Suzuki, Naoki Yagi, Shunsaku Furuke, Takashi Ooki, Ryusuke Aihara, Akira Mogi, Yasuo Hosouchi, Ken Shirabe

**Affiliations:** 1 Department of Surgery, Gunma Prefecture Saiseikai Maebashi Hospital, 564-1, Kamishinden-Machi, Maebashi, Gunma 371-0821 Japan; 2 Division of Hepatobiliary and Pancreatic Surgery, Department of General Surgical Science, Graduate School of Medicine, Gunma University, 3-39-22, Showa-Machi, Maebashi, Gunma 371-8511 Japan

**Keywords:** Primary duodenal carcinoma, Ectopic gastric mucosa, Duodenal stenosis

## Abstract

**Background:**

Ectopic gastric mucosa mainly occurs in the duodenal bulb, and its etiology is thought to be congenital straying of gastric tissues. Primary duodenal carcinoma is a rare disease; however, reports of carcinoma arising from ectopic gastric mucosa are extremely rare. We report a case of primary duodenal carcinoma suspected to arise from ectopic gastric mucosa, which discovered as a result of duodenal stenosis.

**Case presentation:**

The patient was a 71-year-old man with persistent weight loss and white stools. Enhanced computed tomography showed stenosis of the third portion of the duodenum and main pancreatic duct dilatation. Upper gastrointestinal endoscopy revealed irregularity of the duodenal mucosa from the anorectal side of the papilla of Vater to the stenosis of the third portion. No malignant cells were found by biopsies from the duodenal mucosa. Endoscopic ultrasonography did not detect the tumor in the pancreatic head. The possibility of a pancreatic tumor could not be ruled out based on findings of main pancreatic duct dilatation in the pancreatic head, and the patient had long-term poor oral intake because of duodenal stenosis; thus, surgical treatment was planned. Intraoperative findings showed palpable induration of the third portion of the duodenum and white nodules on the serosal surface. This was diagnosed as primary duodenal carcinoma, and pylorus-preserving pancreatoduodenectomy was performed. Histopathological diagnosis revealed ectopic gastric mucosa in the papilla of Vater and well-differentiated tubular adenocarcinoma invaded the normal duodenal submucosa and extended to the duodenal serosa. No mass lesion was detected in the pancreas, and an intraductal papillary mucinous neoplasm was observed in the branch pancreatic duct. The main pancreatic duct stricture was caused by the duodenal carcinoma invasion.

**Conclusions:**

This case of primary duodenal carcinoma was suspected to arise from ectopic gastric mucosa and review the relevant literature.

## Background

Ectopic gastric mucosa mainly occurs in the duodenal bulb, and its etiology is thought to be congenital straying of gastric tissues. Primary duodenal carcinoma is a rare disease; however, reports of carcinoma arising from ectopic gastric mucosa are extremely rare. Herein, we present a case of primary duodenal carcinoma suspected to arise from ectopic gastric mucosa, which discovered as a result of duodenal stenosis.

## Case presentation

A 71-year-old man complained of loss of appetite 6 months before his visit to our hospital, had lost 9 kg of weight in 2 months, and had white stools, so he was brought to a referring physician. The patient was referred to our hospital for careful examination and treatment. He had no past history or family history of malignancy. He had a drinking habit and a smoking habit of approximately 15 cigarettes per day.

At the initial visit, he complained of loss of appetite and physical weariness. Blood tests were normal, including carcinoembryonic antigen (CEA) of 2.4, IgG4 of 51.1. The carbohydrate antigen 19-9 (CA 19–9) mildly elevated of 49.7.

**Fig. 1:**
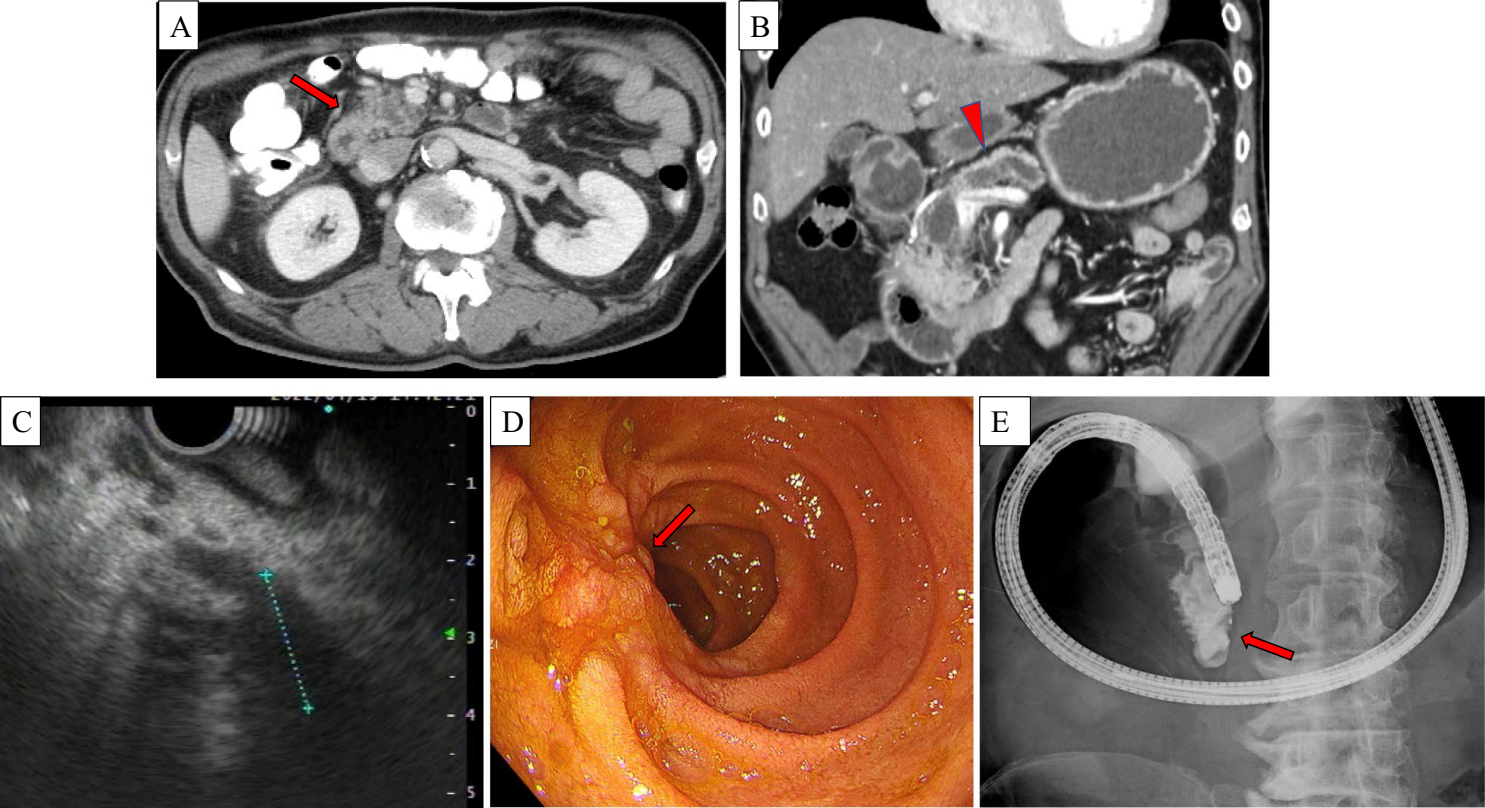
Enhanced abdominal CT and GI endoscopic findings. Enhanced CT showed stenosis in the third portion of the duodenum in the (**A**) (red arrow) axial view and (**B**) (red arrow) coronal view. Enhanced CT also revealed dilatation of the main pancreatic duct (**B**) (red arrowhead) and atrophy of the pancreatic head. EUS revealed a hypoechoic area at the deep lesion of pancreatic head, approximately 20 mm (**C**). GI endoscopy revealed one-third circumferential mucosal irregularity from the anal side of the main duodenal papilla to the inferior duodenal angulus, which bled easily (**D**) (red arrow). A biopsy was performed from the same site. Severe stenosis was observed from the inferior duodenal angulus to the third portion, and the scope could not pass through (**E**) (red arrow)

Enhanced computed tomography (CT) revealed the stenosis in the third portion of the duodenum, main pancreatic duct dilatation, and atrophy of the pancreatic head (Fig. 1A, B).

Upper Gastrointestinal (GI) endoscopy was also performed, which revealed one-third circumferential mucosal irregularity from the anal side of the main duodenal papilla to the inferior duodenal angulus, which bled easily (Fig. 1C). A biopsy was performed from the same site. Severe stenosis was observed from the inferior duodenal angulus to the third portion, and the scope could not pass through it. The contrast medium also could not pass through the stenosis and refluxed into the stomach (Fig. 1D). The biopsy revealed inflamed duodenal mucosa, and no malignant cells were found.

As described above, the initial findings of examinations were suspicious for duodenal stenosis caused by duodenal tumor or pancreatic head tumor. Moreover, endoscopic ultrasonography (EUS) was performed for close examination. The main pancreatic duct was stenosed at the pancreatic head, which measured approximately 10 mm in diameter at the transition area of the pancreatic head body. A hypoechoic area at the deep lesion of pancreatic head, approximately 20 mm was suspected at the narrowing of the main pancreatic duct, but tumor evaluation was difficult. We considered performing endoscopic ultrasound-guided fine needle aspiration (EUS–FNA) on the same area; however, the presence of the artifact made it difficult to perform a detailed evaluation. Furthermore, the stenosis at the third portion of the duodenum made it difficult to secure a safe surgical field for puncture. Therefore, EUS–FNA was not feasible owing to technical reasons.

Although preoperative histological diagnosis was impossible, we diagnosed pancreatic head cancer or duodenal cancer based on imaging findings. Regarding the treatment plan, as the patient was predicted to have advanced cancer based on the results of preoperative enhanced CT and GI endoscopy and the patient continued to have difficulty with oral intake due to duodenal stenosis, we decided to perform pylorus-preserving pancreatoduodenectomy (PPPD) as a diagnostic treatment.

**Fig. 2:**
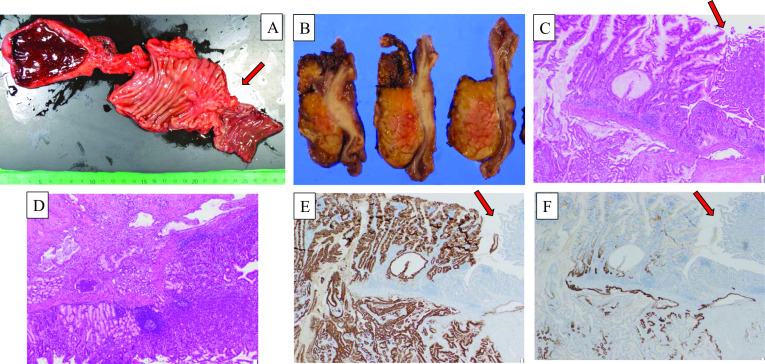
Histopathological findings and immunohistochemical staining test. The resected specimen showed a 40 × 30 mm tumor on the descending part of the duodenum slightly more distal to the papilla of Vater, and a white thickening of the duodenal wall was observed on the sectioned surface of the same area (**A**, **B**) (red arrow). There was an infiltrative growth of carcinoma cells with a predominant component of well to poorly differentiated tubular adenocarcinoma under the normal duodenal mucosa (**C**, **D**). The non-tumor duodenal epithelium is observed in the upper right corner of this image (red arrow), showing the continuous infiltration of the intramucosal hypodense lesion into the submucosal layer of the duodenal epithelium (**C**). Cancer cells were developing under the duodenal submucosa and infiltrated into the duodenal muscularis propria (**D**). The immunostaining of the same area showed that MUC5AC, which stained the gastric crypt epithelium, was diffusely positive in the intramucosal and tumor-infiltrated area, whereas MUC6 was negative to partially positive in the mucosa and positive in approximately 50% of the infiltrated area (**E**, **F**)

Intraoperative findings: Upon opening the abdomen, abdominal cavity had no obvious nodules or ascites. Liver metastases and peritoneal dissemination were absent, as confirmed by detailed intraperitoneal observation. Initially, rapid peritoneal lavage cytology was performed, and the results were negative. A firm mass was palpable on the third duodenal portion, and a white nodule was observed on the serosal surface in a partially distal direction. Visual examination showed that duodenal cancer was the more likely diagnosis. Moreover, no unresectable component was noted at that point. PPPD was conducted as scheduled without performing intraoperative rapid pathology diagnosis. The mesentery of the transverse colon was partially resected. No obvious lymph node enlargement was noted. PPPD, D2 Lymph node dissection, and modified Child procedure (operation time, 5 h 44 min; amount of bleeding, 610 ml) was performed without problem. The resected specimen showed a 40 × 30 mm tumor on the descending part of the duodenum slightly more distal to the papilla of Vater, and a white thickening of the duodenal wall was observed on the sectioned surface of the same area (Fig. 2A).

Histopathological findings: Primary duodenal carcinoma, pT4, pN2(#12b,#17a,#17b), cM0, pStage IIIB (Union for International Cancer Control 8^th^ edition), intraductal papillary mucinous neoplasm (IPMN).

A 14-mm flat prominence was protruding into the lumen on the distal side of the papilla of Vater and tubular development similar to the gastroduodenal gland. On the same area, there was an infiltrative growth of carcinoma cells with a predominant component of well to poorly differentiated tubular adenocarcinoma under the normal duodenal mucosa (Fig. 2B, C). The non-tumor duodenal epithelium is observed in the upper right corner of the image, showing the continuous infiltration of the intramucosal hypodense lesion into the submucosal layer of the duodenal epithelium (Fig. 2B). In addition, immunostaining of the same area showed that MUC5AC, which stained the gastric crypt epithelium, was diffusely positive in the intramucosal and tumor-infiltrated area, whereas MUC6 was negative to partially positive in the mucosa and positive in approximately 50% of the infiltrated area. (Fig. 2D, E). MUC2 was negative in all cases. These pathological findings indicated the presence of a transition zone between ectopic gastric mucosa and normal mucosa in the duodenal lesion. No malignancy was found in the pancreas, and cancerous infiltration was observed around the papilla of Vater, but not around the stenotic area of the main pancreatic duct. On the contrary, an IPMN was noted in the branch pancreatic duct, which was covered with low-grade to high-grade columnar epithelium. Based on the EUS findings, the hypoechoic region was drawn at the deep lesion of the pancreatic head. Moreover, EUS may have drawn the duodenal carcinoma in the third portion beyond the pancreatic head, as noted from a retrospective view. No cancer cells were detected in the stenosis area of the main pancreatic duct during pathological examination. Pathologically, the cause of the main pancreatic duct stenosis was not duodenal carcinoma or pancreatic head carcinoma. The apparent cause remains unclear. Intraoperative findings showed that the pancreas itself was hard, and the cause may have been spillover of inflammation caused by duodenal stenosis. Therefore, this case was considered primary duodenal carcinoma arising from ectopic gastric mucosa.

Postoperative course: The patient resumed oral intake on postoperative day 5, and the intraperitoneal drains were removed sequentially on postoperative day 8. He was discharged home on postoperative day 29 without any problem. On postoperative day 38, the drug combination TS-1 (tegafur/gimeracil/oteracil potassium), an oral 5-FU drug, was started as adjuvant chemotherapy. The patient continues to receive treatment without adverse events, and the CA19-9 values were 13.5 and a10.0 at 2 and 6 months postoperatively, respectively. He is alive and recurrence-free 7 months after surgery.

## Discussion

Primary duodenal carcinoma is reported to occur in 3.0–3.7 per million populations in North America, and the incidence rate is slowly increasing. In Japan, a high incidence rate of 23.7 per million populations was reported [1]. However, the frequency of primary duodenal carcinoma in the entire gastrointestinal tract is reported to be 0.3%, making it one of the rarest gastrointestinal tract cancers [2]. On the contrary, the frequency of the detection of duodenal ectopic gastric mucosa by upper GI endoscopy is approximately 1.0% [3]; however, ectopic gastric mucosa is frequently identified in duodenal elevated lesions, especially in the duodenal bulb at 11% [4], making it an important differential disease. Ectopic gastric mucosa of the duodenum is narrowly defined as the presence of gastric fundic glandular tissue and gastric-type coated epithelium, and its etiology is thought to be congenital straying of the gastric fundus glands [5]. Ectopic gastric mucosa of the duodenum is carcinogenic; a Japanese report showed that 3.4% of early carcinomas of the duodenal bulb arise from ectopic gastric mucosa [6]. The mechanism of carcinomatosis is considered the metaplasia–dysplasia–carcinoma sequence or adenoma–carcinoma sequence, as in other epithelial tissues. However, the frequency of occurrence and number of reported cases suggest a statistically low frequency of carcinomatosis [7]. Thus, ectopic gastric mucosa is detected and screened relatively frequently as the accuracy of upper GI endoscopy improves. However, it is recognized as a benign disease in itself, and its relationship to carcinogenesis is controversial. In the presented case, although no microscopically obvious gastric fundus gland structure was observed, histopathologically, ectopic gastric mucosa and cancer cells coexisted in the tumor area, and the transition zone between the ectopic gastric mucosa and cancer cells was identified (Fig. 2B, C). Moreover, the immunohistochemical staining test for tumor area was positive for MUC5AC and MUC6, also its pattern was different from that of normal duodenal mucosa. These pathological findings indicated the presence of a transition zone between ectopic gastric mucosa and normal mucosa in the duodenal lesion. Therefore, we diagnosed this case as duodenal carcinoma arising from ectopic gastric mucosa. In Japan, most duodenal carcinomas that arise from ectopic gastric mucosa are early stage carcinomas [8, 9], and there have been no reports of advanced carcinomas, such as our advanced case, which was diagnosed due to the obstruction of the passage caused by duodenal stenosis.

Moreover, this case is this case is rare considering that it occurs in the third portion of the duodenum. Kiyomori et al. reported on duodenal tumors with gastric epithelium in the gastrointestinal tract and their site of origin [10]. Among 35 lesions, four lesions (11%) originated on the anorectal side of the papilla of Vater. In addition, although all tumors were early stage carcinomas, only one of six duodenal carcinomas was located on the anorectal side of the papilla of Vater. Among them, none occurred on the third portion of the duodenum. From an embryological point of view, ectopic gastric mucosa in the third portion of the duodenum is considered to be a rare occurrence. Bordered by the papilla of Vater, the oral side is of foregut origin, whereas the anorectal side, including the third portion, is of midgut origin. Since the stomach is derived from the foregut, stray gastric fundic glands may occur during development in the duodenum on the oral side, including the duodenal bulb, resulting in ectopic gastric mucosa. Owing to the fact that the mechanism of ectopic gastric mucosa is the congenital straying of gastric fundic glands, the frequency of ectopic gastric mucosa is extremely rare in the third portion, which has a different embryological classification from the stomach.

As mentioned above, ectopic gastric mucosa in the duodenum is frequently detected by upper GI endoscopy, which is characterized by scattered small hemispherical ridges and clusters of flattened ridges. Magnifying endoscopy and narrow band imaging (NBI) of the gastric mucosa shows a gastric small furrow pattern that closely resembles the gastric crypt epithelium with little villous structure, which is considered useful for diagnosis [10]. Accurate diagnosis by biopsy is often difficult. Duodenal carcinoma often presents as an epithelial elevated lesion, whereas tumors arising from ectopic gastric mucosa often present as a partially submucosal tumor-like elevation. It is reported that an aperture is exist at the apex of the tumor, and an epithelial component consisting of gastric orbital epithelium is present at the same site [6]. In the present case, despite multiple biopsies, no malignant findings were obtained, which was attributed to the morphological characteristics of the cancer arising from ectopic gastric mucosa. The histopathological diagnosis also showed a proliferative infiltration of cancer cells in the central part of the ectopic gastric mucosa, with findings of cancer cells extending, i.e., developing under the duodenal submucosa covered by the normal mucosa and spreading from the intrinsic muscular layer to the serosal surface. This finding also suggests that no cancer cells were exposed on the mucosal surface; thus, the biopsy forceps may have failed fully grasp the epithelial tissue with cancer cells. Although tissue biopsy is essential for the diagnosis of malignancy, careful biopsy should be performed with these morphological features in mind when endoscopic findings of ectopic gastric mucosa are suspected. When suspecting a cancer arising from ectopic gastric mucosa, for an accurate diagnosis, comprehensive evaluation of imaging findings, including tumor markers and blood tests, endoscopic macroscopic evaluation, and correct tissue biopsy are necessary.

Endoscopic treatment including endoscopic mucosal resection (EMR) and endoscopic submucosal dissection (ESD) or surgical resection is the treatment of choice, and to evaluate tumor progression, ultrasound endoscopy is important in determining the treatment strategy. Regarding the relationship between tumor progression and lymph node metastasis in primary duodenal cancer, a study reported that lymph node metastasis is not observed in mucosal cancer, 5–11% in submucosal cancer, and more frequently in cancers deeper than the muscularis propria [11]. Endoscopic treatment such as EMR and ESD are considered for mucosal cancer, but they are technically difficult because of the anatomical difficulty in securing the field of view, and the incidence of gastrointestinal perforation and posterior hemorrhage is still higher than that of endoscopic treatment for other organs; thus, they are only weakly recommended, because the procedure should be performed by surgeons skilled in the technique and at the appropriate facility [1]. In addition, duodenal carcinomas arising from ectopic gastric mucosa are likely to be deeper than the submucosa, considering the aforementioned submucosal tumor-like invasive morphology. This suggests that surgical treatment should be selected as the standard treatment. As mentioned above, lymph node metastasis is observed when duodenal cancer has progressed deeper than the submucosal layer, and the frequency of lymph node metastasis increases as the degree of progression increases. Considering these tumor factors, pancreatoduodenectomy is currently the standard procedure for duodenal cancer in the submucosal layer or deeper. On the contrary, the 5-year postoperative survival rates of pancreaticoduodenectomy and local resection of the duodenum for duodenal cancer are comparable, and some reports have indicated the possibility and appropriateness of so-called reduced surgery such as local resection of the duodenum because of the low incidence of perioperative complications and low invasiveness [12]. Therefore, a reduced surgery may be considered for mucosal carcinoma. However, for duodenal carcinoma arising from ectopic gastric mucosa, pancreaticoduodenectomy should still be selected based on the type of cancer invasion, followed by lymph node dissection. According to reports from Japan, the frequency of lymph node metastasis was 11.4% in subpyloric lymph nodes (No. 6), 5.7% in portal vein lymph nodes (No. 12p), 8.6% in the lymph nodes around the common hepatic artery (No. 8ap), 17.1% in posterior pancreatic head lymph nodes (No. 13ab), 22.7% in anterior pancreatic head lymph nodes (No. 17 ab), 22.9% in the peri-SMA (No.14pd), and 5.7% in the peri-aortic [13]. This suggests that pancreatoduodenectomy with peripancreatic lymph node dissection is reasonable. In our case, although duodenal stenosis made it difficult to accurately assess tumor progression by EUS, preoperative imaging evaluation, including fluoroscopic endoscopy and enhanced CT, clearly revealed that it was advanced cancer with submucosal invasion or deeper. Moreover, the possibility of a malignant tumor of the pancreatic head due to the dilatation of the main pancreatic duct prompted us to perform PPPD and lymph node dissection according to the procedure for pancreatic head cancer. Histopathological examination of the excised specimen revealed that the tumor was exposed on the serosal surface beyond the submucosal layer. Regarding lymph node metastasis, three lymph nodes were positive, including the peri-bile duct lymph node (No. 12b) and the anterior pancreatic head lymph node (No. 17ab). The pathological diagnosis was pT4, pN2, cM0, pStage IIIB, and it was advanced duodenal carcinoma with positive regional lymph nodes. Given that positive lymph nodes are one of the poor prognostic factors in duodenal carcinoma [14], we believe that lymph node dissection according to pancreatic head cancer was appropriate. However, no prospective studies have examined the significance of lymph node dissection and the optimal extent of lymph node dissection; thus, future validation is needed.

There is a possibility that it increases the options of adjuvant chemotherapy from the diagnosis of the carcinoma arising from ectopic gastric mucosa; thus, even if the pathological examination in this case was done postoperatively, the presence of ectopic gastric mucosa should be revealed, and a detailed histopathological examination including immunohistochemistry staining test is important; thus, even if the pathological examination in this case was done postoperatively, the presence of ectopic gastric mucosa should be revealed, and a detailed histopathological examination including immunohistochemistry staining test is necessary. Although esophageal carcinoma is the most frequently reported case of carcinoma arising from ectopic gastric mucosa, the number of cases is small. Chemotherapy regimens similar to those used for primary organ or gastric cancer are considered effective; however, there is no consensus on treatment strategy. On the contrary, no randomized controlled trials have compared postoperative adjuvant chemotherapy with surgery alone for duodenal carcinoma, and the efficacy of adjuvant chemotherapy is controversial. A clinical trial (JCOG1502C; J-BALLAD study) is currently underway in Japan to evaluate the superiority of postoperative capecitabine plus oxaliplatin over surgery alone, and the results of this trial are awaited.

This patient had a pStage III case who had undergone curative resection, and postoperative adjuvant chemotherapy with TS-1 was selected according to the histopathology of the gastric cancer. In general, for the advanced gastric cancer (e.g., greater than pStageIII), combined chemotherapy such as TS1 + oxaliplatin is indicated as postoperative adjuvant chemotherapy. However, due to the patient's poor general condition including poor nutrition and weight loss because of long-term poor oral intake before surgery, we judged that combined chemotherapy would be too invasive for the patient and chose TS-1 alone as postoperative adjuvant chemotherapy.

Despite being alive and recurrence-free 7 months after surgery, the chemotherapy regimen should be similar to that for gastric cancer even at the time of recurrence.

## Conclusion

We present a case of primary duodenal carcinoma suspected to arise from ectopic gastric mucosa, which was discovered with duodenal stenosis. This is an extremely rare case, and no studies have reported advanced cases like this one. As this is a case of cancer from an ectopic gastric mucosa, the selection of multidisciplinary treatment including postoperative adjuvant chemotherapy will require further examination based on the accumulation of cases in the future.

## Data Availability

Data sharing is not applicable to this article as no data sets were generated or analyzed during the current study.

## Abbreviations

CT: Computed tomography
EUS: Endoscopic ultrasonography
EUS–FNA: Endoscopic ultrasound-guided fine needle aspiration
GI endoscopy: Gastrointestinal endoscopy
PPPD: Pylorus-preserving pancreatoduodenectomy
IPMN: Intraductal papillary mucinous neoplasm
EMR: Endoscopic mucosal resection
ESD: Endoscopic submucosal dissection
